# Tris(1,10-phenanthroline-κ^2^ *N*,*N*′)iron(II) bis­[(1,10-phenanthroline-κ^2^ *N*,*N*′)tetra­kis­(thio­cyanato-κ*N*)chromate(III)] acetonitrile tris­olvate monohydrate

**DOI:** 10.1107/S1600536812012949

**Published:** 2012-04-04

**Authors:** Valentyna V. Semenaka, Oksana V. Nesterova, Volodymyr N. Kokozay, Volodymyr V. Bon

**Affiliations:** a Department of Inorganic Chemistry, Taras Shevchenko National University of Kyiv, 64 Volodymyrs’ka St., Kyiv 01601, Ukraine; b Department of Chemistry of Complex Compounds, V.I. Vernadsky Institute of General and Inorganic Chemistry, National Academy of Sciences of Ukraine, 32/34 Palladin Ave., Kyiv 03680, Ukraine

## Abstract

Single crystals of the title heterometallic compound, [Fe(C_12_H_8_N_2_)_3_][Cr(NCS)_4_(C_12_H_8_N_2_)]_2_·3CH_3_CN·H_2_O or [Fe(Cphen)_3_][Cr(NCS)_4_(phen)]_2_·3CH_3_CN·H_2_O, were pre­pared using the one-pot open-air reaction of iron powder, Reineckes salt and 1,10-phenanthroline (phen) in acetonitrile. The asymetric unit consists of an [Fe(phen)_3_]^2+^ cation, two [Cr(phen)(NCS)_4_]^−^ anions, three acetonitrile solvent mol­ecules and a water mol­ecule. The Fe and Cr atoms both show a slightly distorted octa­hedral FeN_6_ and CrN_6_ coordination geometry with adjacent angles in the range 79.67 (12)–95.21 (12)°. No classical hydrogen bonding involving the water molecule is observed.

## Related literature

For background to direct synthesis, see: Makhankova (2011 ▶). For background to the use of Reineckes salt as a source of building blocks or metalloligands, see: Zhang *et al.* (2001 ▶); Cucos *et al.* (2006 ▶); Cherkasova & Gorunova (2003 ▶); Nikitina *et al.* (2009 ▶); Kolotilov *et al.* (2010 ▶). For Fe—N bond lengths in iron–phen derivatives, see: Alonso *et al.* (2005 ▶). For a related structure, see: Semenaka *et al.* (2011 ▶). 
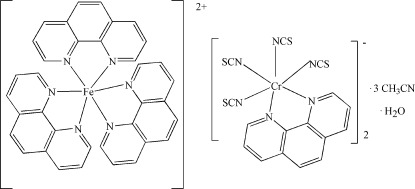


## Experimental

### 

#### Crystal data

[Fe(C_12_H_8_N_2_)_3_][Cr(NCS)_4_(C_12_H_8_N_2_)]_2_·3C_2_H_3_N·H_2_O
*M* *_r_* = 1666.69
Triclinic, 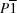

*a* = 12.641 (2) Å
*b* = 16.681 (3) Å
*c* = 20.692 (4) Å
α = 111.514 (5)°
β = 107.291 (6)°
γ = 92.231 (5)°
*V* = 3821.5 (13) Å^3^
*Z* = 2
Mo *K*α radiation
μ = 0.75 mm^−1^
*T* = 173 K
0.23 × 0.22 × 0.12 mm

#### Data collection

Bruker APEXII CCD diffractometer
Absorption correction: multi-scan (*SADABS*; Bruker, 2005 ▶) *T*_min_ = 0.847, *T*_max_ = 0.916
48046 measured reflections
15519 independent reflections
8993 reflections with *I* > 2σ(*I*)
*R*_int_ = 0.078

#### Refinement

*R*[*F* ^2^ > 2σ(*F* ^2^)] = 0.051
*wR*(*F* ^2^) = 0.135
*S* = 0.99
15519 reflections
974 parameters
3 restraints
H atoms treated by a mixture of independent and constrained refinement
Δρ_max_ = 0.43 e Å^−3^
Δρ_min_ = −0.44 e Å^−3^

### 

Data collection: *APEX2* (Bruker, 2005 ▶); cell refinement: *SAINT* (Bruker, 2005 ▶); data reduction: *SAINT*; program(s) used to solve structure: *SHELXS97* (Sheldrick, 2008 ▶); program(s) used to refine structure: *SHELXS97* (Sheldrick, 2008 ▶); molecular graphics: *PLATON* (Spek, 2009 ▶); software used to prepare material for publication: *publCIF* (Westrip, 2010 ▶).

## Supplementary Material

Crystal structure: contains datablock(s) I, global. DOI: 10.1107/S1600536812012949/ru2031sup1.cif

Supplementary material file. DOI: 10.1107/S1600536812012949/ru2031Isup2.cdx

Structure factors: contains datablock(s) I. DOI: 10.1107/S1600536812012949/ru2031Isup3.hkl

Additional supplementary materials:  crystallographic information; 3D view; checkCIF report

## Figures and Tables

**Fig. 1.**
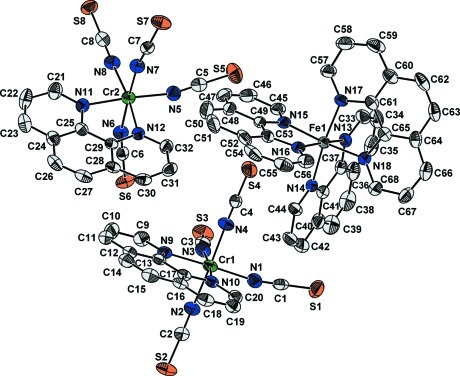
Crystal structure of the complex, showing the atom numbering, with 50% probability displacement ellipsoids. Solvent molecules are omitted for clarity.

## supplementary crystallographic information

### Comment

Recently we have reported that the Reineckes salt, (NH)_4_[Cr(NCS)_4_(NH_3_)_2_]^.^H_2_O, could be exploited as a source of building blocks or metalloligands in the direct synthesis of heterometallic Cu/Cr and Co/Cr compounds with amines and Schiff-base ligands (Nikitina *et al.*., 2009; Semenaka *et al.*., 2011). In the present work, we report that the reaction of iron powder, Reineckes salt and 1,10-phenanthroline (phen) in acetonitrile solution has afforded a single crystals of the novel heterometallic Fe/Cr compound. The asymetric unit of title compound consists of slightly distorted octahedral [Fe(phen)_3_]^2+^ cation and two [Cr(phen)(NCS)_4_]^-^ anion blocks as well as solvate acetonitrile and water molecules (Fig. 1). In complex cation iron centers are in elongated octahedral coordination environment with six nitrogen atoms phen ligands. The bond lengths of Fe–N vary in the range 1,959 (3) – 1,980 (3) Å which is in the range usually found in other iron-phen derivatives (Alonso *et al.*, 2005). The *cis* and *trans* N–Fe–N bond angles vary from 82,27 (12)° to 95.21 (12)° and from 172.90 (12)° to 174.04 (11)°, respectively. The Cr(III) ions have N6 donor set formed by two N atoms from phen, which are replaced NCS groups in initial anions of Reineckes salt, and two NCS-groups in the equatorial plane and by two another NCS-groups at the axial positions. The axial Cr–N bond lengths are vary from 1,962 (3) to 2,077 (3) Å. The *cis* and *trans* N–Cr–N bond angles vary from 79,80 (11)° to 94,97 (12)° and from 170,61 (12)° to 174,77 (12)°, respectively. The thiocyanate groups are almost linear, bond angles N–C–S are in a narrow range: from 178.1 (3) ° to 179.3 (4)°.

### Experimental

Iron powder (0.07 g, 1.25 mmol), NH_4_[Cr(NCS)_4_(NH_3_)_2_]^.^H_2_O (0.443 g, 1.25 mmol), NH_4_NCS (0.190 g, 2.5 mmol), phen^.^H_2_O (0.496 g, 2.5 mmol) and acetonitrile (15 ml) were heated to 50–60° and stirred magnetically until total dissolution of the iron was observed (4,5 h). The resulting red solution was slowly evaporated at room temperature until dark-red crystals suitable for crystallographic study were formed. The crystals were filtered off, washed with dry Pr^i^OH and finally dried *in vacuo* at room temperature. Yield: 0.25 g. IR (KBr, cm^-1^): 3048(sh), 3044(sh), 2314(w), 2066(*vs*), 1996(sh), 1645(sh), 1638(sh), 1635(*m*), 1599(*m*), 1578(*m*), 1554(sh), 1536(w), 1521(*m*), 1494(sh), 1472(sh), 1455(sh), 1424(*s*), 1409(sh), 1373(w), 1307(w), 1271(w), 1256(sh), 1223(sh), 1207(w), 1144(*m*), 1102(*m*), 1057(sh), 1039(sh), 990(*m*), 960(w), 942(sh), 915(sh), 876(w), 870(sh), 843(*s*), 798(*m*), 771(w), 737(sh), 719(*s*), 668(sh), 656(w), 620(w), 581(sh), 557(w), 626(w), 568(sh), 481(*m*), 451(sh), 433(w), 427(sh). The compound is sparingly soluble in dmso and dmf, insoluble in water and it is indefinitely stable in air.

### Refinement

All non-hydrogen atoms were located from the initial solution and refined with anisotropic thermal parameters. The hydrogen atoms were positioned geometrically and included into refinement using riding model approximation with *U*_iso_ = *nU*_eq_ of non-hydrogen carrier atom (*n* = 1.5 for CH_3_ groups and *n* = 1.2 for remaining H-atoms)

### Crystal data

**Table:**

[Fe(C_12_H_8_N_2_)_3_][Cr(NCS)_4_(C_12_H_8_N_2_)]_2_·3C_2_H_3_N·H_2_O	*Z* = 2
*M**_r_* = 1666.69	*F*(000) = 1704
Triclinic, *P*1	*D*_x_ = 1.448 Mg m^−^^3^
*a* = 12.641 (2) Å	Mo *K*α radiation, λ = 0.71073 Å
*b* = 16.681 (3) Å	Cell parameters from 4485 reflections
*c* = 20.692 (4) Å	θ = 2.3–21.7°
α = 111.514 (5)°	µ = 0.75 mm^−^^1^
β = 107.291 (6)°	*T* = 173 K
γ = 92.231 (5)°	Block, red
*V* = 3821.5 (13) Å^3^	0.23 × 0.22 × 0.12 mm

### Data collection

**Table:**

Bruker APEXII CCD diffractometer	15519 independent reflections
Radiation source: fine-focus sealed tube	8993 reflections with *I* > 2σ(*I*)
Graphite monochromator	*R*_int_ = 0.078
φ and ω scans	θ_max_ = 26.4°, θ_min_ = 1.1°
Absorption correction: multi-scan (*SADABS*; Bruker, 2005)	*h* = −15→15
*T*_min_ = 0.847, *T*_max_ = 0.916	*k* = −20→20
48046 measured reflections	*l* = −25→25

### Refinement

**Table:**

Refinement on *F*^2^	Primary atom site location: structure-invariant direct methods
Least-squares matrix: full	Secondary atom site location: difference Fourier map
*R*[*F*^2^ > 2σ(*F*^2^)] = 0.051	Hydrogen site location: inferred from neighbouring sites
*wR*(*F*^2^) = 0.135	H atoms treated by a mixture of independent and constrained refinement
*S* = 0.99	*w* = 1/[σ^2^(*F*_o_^2^) + (0.0572*P*)^2^ + 0.0054*P*] where *P* = (*F*_o_^2^ + 2*F*_c_^2^)/3
15519 reflections	(Δ/σ)_max_ < 0.001
974 parameters	Δρ_max_ = 0.43 e Å^−^^3^
3 restraints	Δρ_min_ = −0.44 e Å^−^^3^

### Special details

**Table:**

Geometry. All e.s.d.'s (except the e.s.d. in the dihedral angle between two l.s. planes) are estimated using the full covariance matrix. The cell e.s.d.'s are taken into account individually in the estimation of e.s.d.'s in distances, angles and torsion angles; correlations between e.s.d.'s in cell parameters are only used when they are defined by crystal symmetry. An approximate (isotropic) treatment of cell e.s.d.'s is used for estimating e.s.d.'s involving l.s. planes.
Refinement. Refinement of *F*^2^ against ALL reflections. The weighted *R*-factor *wR* and goodness of fit *S* are based on *F*^2^, conventional *R*-factors *R* are based on *F*, with *F* set to zero for negative *F*^2^. The threshold expression of *F*^2^ > σ(*F*^2^) is used only for calculating *R*-factors(gt) *etc*. and is not relevant to the choice of reflections for refinement. *R*-factors based on *F*^2^ are statistically about twice as large as those based on *F*, and *R*- factors based on ALL data will be even larger.

### Fractional atomic coordinates and isotropic or equivalent isotropic displacement parameters (Å^2^)

**Table:**

	*x*	*y*	*z*	*U*_iso_*/*U*_eq_	
Cr1	0.27228 (5)	0.11974 (4)	0.24479 (4)	0.03091 (17)	
Cr2	0.65438 (5)	0.31815 (4)	0.08754 (3)	0.02873 (16)	
Fe1	0.21655 (4)	0.67016 (4)	0.34300 (3)	0.02632 (14)	
S1	0.27329 (12)	0.22111 (9)	0.49075 (7)	0.0579 (4)	
S2	0.09452 (10)	−0.17085 (8)	0.16961 (8)	0.0536 (3)	
S3	0.62676 (10)	0.06152 (8)	0.35925 (7)	0.0531 (3)	
S4	0.37743 (12)	0.41344 (8)	0.28415 (7)	0.0530 (3)	
S5	0.77068 (12)	0.50790 (9)	0.33991 (7)	0.0604 (4)	
S6	0.65109 (9)	0.07691 (8)	0.15795 (7)	0.0441 (3)	
S7	0.62707 (10)	0.59283 (8)	0.07321 (7)	0.0491 (3)	
S8	1.02840 (9)	0.36578 (8)	0.08940 (7)	0.0451 (3)	
N1	0.2488 (3)	0.1575 (2)	0.3417 (2)	0.0376 (9)	
N2	0.2035 (3)	−0.0022 (2)	0.21591 (19)	0.0371 (9)	
N3	0.4240 (3)	0.0943 (2)	0.28350 (18)	0.0356 (8)	
N4	0.3298 (3)	0.2410 (2)	0.26495 (18)	0.0358 (8)	
N5	0.6900 (3)	0.3873 (2)	0.1943 (2)	0.0371 (9)	
N6	0.6551 (3)	0.2076 (2)	0.10439 (18)	0.0346 (8)	
N7	0.6422 (3)	0.4265 (2)	0.07093 (18)	0.0341 (8)	
N8	0.8136 (3)	0.3295 (2)	0.09225 (18)	0.0354 (8)	
N9	0.2783 (3)	0.0844 (2)	0.13939 (18)	0.0309 (8)	
N10	0.1155 (3)	0.1396 (2)	0.19199 (17)	0.0285 (7)	
N11	0.6017 (3)	0.2448 (2)	−0.02542 (17)	0.0300 (8)	
N12	0.4819 (3)	0.2997 (2)	0.06400 (17)	0.0277 (7)	
N13	0.3234 (3)	0.6688 (2)	0.43423 (17)	0.0318 (8)	
N14	0.1557 (3)	0.5523 (2)	0.33057 (17)	0.0277 (7)	
N15	0.3167 (3)	0.6249 (2)	0.28571 (18)	0.0304 (8)	
N16	0.1125 (3)	0.6567 (2)	0.24675 (18)	0.0303 (8)	
N17	0.2766 (3)	0.7928 (2)	0.36711 (18)	0.0318 (8)	
N18	0.1187 (3)	0.7270 (2)	0.39894 (17)	0.0294 (8)	
C1	0.2574 (3)	0.1841 (3)	0.4040 (3)	0.0363 (10)	
C2	0.1576 (3)	−0.0722 (3)	0.1971 (2)	0.0335 (10)	
C3	0.5093 (3)	0.0804 (3)	0.3150 (2)	0.0363 (10)	
C4	0.3482 (3)	0.3134 (3)	0.2722 (2)	0.0304 (9)	
C5	0.7241 (3)	0.4381 (3)	0.2550 (3)	0.0359 (10)	
C6	0.6516 (3)	0.1528 (3)	0.1264 (2)	0.0298 (9)	
C7	0.6361 (3)	0.4963 (3)	0.0724 (2)	0.0294 (9)	
C8	0.9035 (3)	0.3456 (3)	0.0913 (2)	0.0323 (10)	
C9	0.3598 (3)	0.0550 (3)	0.1145 (2)	0.0423 (11)	
H9	0.4260	0.0487	0.1478	0.051*	
C10	0.3528 (4)	0.0328 (3)	0.0414 (3)	0.0476 (12)	
H10	0.4130	0.0112	0.0254	0.057*	
C11	0.2582 (4)	0.0423 (3)	−0.0073 (2)	0.0411 (11)	
H11	0.2530	0.0281	−0.0572	0.049*	
C12	0.1700 (3)	0.0730 (3)	0.0169 (2)	0.0346 (10)	
C13	0.1840 (3)	0.0932 (2)	0.0913 (2)	0.0301 (9)	
C14	0.0672 (4)	0.0842 (3)	−0.0288 (2)	0.0390 (11)	
H14	0.0572	0.0716	−0.0792	0.047*	
C15	−0.0159 (3)	0.1123 (3)	−0.0024 (2)	0.0382 (11)	
H15	−0.0832	0.1192	−0.0343	0.046*	
C16	−0.0044 (3)	0.1321 (2)	0.0735 (2)	0.0315 (10)	
C17	0.0967 (3)	0.1231 (2)	0.1195 (2)	0.0279 (9)	
C18	−0.0874 (3)	0.1580 (3)	0.1043 (2)	0.0362 (10)	
H18	−0.1578	0.1636	0.0746	0.043*	
C19	−0.0685 (3)	0.1754 (3)	0.1769 (2)	0.0379 (10)	
H19	−0.1248	0.1944	0.1982	0.045*	
C20	0.0349 (3)	0.1652 (2)	0.2197 (2)	0.0344 (10)	
H20	0.0475	0.1769	0.2702	0.041*	
C21	0.6622 (4)	0.2196 (3)	−0.0695 (2)	0.0428 (11)	
H21	0.7415	0.2367	−0.0487	0.051*	
C22	0.6150 (4)	0.1688 (3)	−0.1451 (3)	0.0525 (13)	
H22	0.6616	0.1519	−0.1747	0.063*	
C23	0.5010 (4)	0.1438 (3)	−0.1760 (3)	0.0482 (12)	
H23	0.4679	0.1079	−0.2271	0.058*	
C24	0.4338 (4)	0.1714 (3)	−0.1322 (2)	0.0360 (10)	
C25	0.4875 (3)	0.2212 (2)	−0.0566 (2)	0.0288 (9)	
C26	0.3135 (4)	0.1526 (3)	−0.1594 (2)	0.0413 (11)	
H26	0.2757	0.1178	−0.2102	0.050*	
C27	0.2526 (4)	0.1829 (3)	−0.1146 (2)	0.0392 (11)	
H27	0.1729	0.1705	−0.1347	0.047*	
C28	0.3064 (3)	0.2338 (2)	−0.0370 (2)	0.0311 (9)	
C29	0.4245 (3)	0.2515 (2)	−0.0088 (2)	0.0263 (9)	
C30	0.2486 (3)	0.2690 (3)	0.0123 (2)	0.0359 (10)	
H30	0.1688	0.2601	−0.0049	0.043*	
C31	0.3074 (3)	0.3161 (3)	0.0850 (2)	0.0388 (11)	
H31	0.2687	0.3394	0.1190	0.047*	
C32	0.4244 (3)	0.3298 (3)	0.1094 (2)	0.0343 (10)	
H32	0.4643	0.3619	0.1604	0.041*	
C33	0.4073 (3)	0.7303 (3)	0.4874 (2)	0.0407 (11)	
H33	0.4205	0.7846	0.4835	0.049*	
C34	0.4762 (4)	0.7181 (3)	0.5483 (2)	0.0498 (13)	
H34	0.5352	0.7634	0.5849	0.060*	
C35	0.4589 (4)	0.6411 (4)	0.5554 (2)	0.0526 (13)	
H35	0.5064	0.6325	0.5966	0.063*	
C36	0.3710 (4)	0.5746 (3)	0.5017 (2)	0.0428 (11)	
C37	0.3056 (3)	0.5923 (3)	0.4424 (2)	0.0316 (9)	
C38	0.3420 (4)	0.4927 (3)	0.5040 (3)	0.0514 (13)	
H38	0.3865	0.4796	0.5436	0.062*	
C39	0.2537 (5)	0.4335 (3)	0.4520 (3)	0.0540 (13)	
H39	0.2361	0.3799	0.4558	0.065*	
C40	0.1863 (4)	0.4501 (3)	0.3911 (2)	0.0407 (11)	
C41	0.2133 (3)	0.5289 (3)	0.3860 (2)	0.0304 (9)	
C42	0.0900 (4)	0.3932 (3)	0.3352 (3)	0.0492 (13)	
H42	0.0659	0.3392	0.3362	0.059*	
C43	0.0321 (4)	0.4165 (3)	0.2800 (2)	0.0456 (12)	
H43	−0.0332	0.3787	0.2424	0.055*	
C44	0.0675 (4)	0.4948 (3)	0.2780 (2)	0.0372 (10)	
H44	0.0272	0.5081	0.2376	0.045*	
C45	0.4222 (3)	0.6122 (3)	0.3077 (3)	0.0388 (11)	
H45	0.4587	0.6254	0.3585	0.047*	
C46	0.4818 (4)	0.5801 (3)	0.2591 (3)	0.0531 (14)	
H46	0.5564	0.5702	0.2768	0.064*	
C47	0.4325 (5)	0.5629 (3)	0.1860 (3)	0.0582 (15)	
H47	0.4731	0.5422	0.1528	0.070*	
C48	0.3220 (4)	0.5761 (3)	0.1604 (3)	0.0462 (12)	
C49	0.2676 (4)	0.6073 (2)	0.2123 (2)	0.0326 (10)	
C50	0.2600 (6)	0.5605 (3)	0.0852 (3)	0.0634 (16)	
H50	0.2954	0.5399	0.0489	0.076*	
C51	0.1544 (6)	0.5741 (3)	0.0648 (3)	0.0615 (16)	
H51	0.1162	0.5625	0.0145	0.074*	
C52	0.0969 (5)	0.6058 (3)	0.1170 (2)	0.0466 (12)	
C53	0.1562 (4)	0.6232 (2)	0.1911 (2)	0.0347 (10)	
C54	−0.0145 (5)	0.6198 (3)	0.1000 (3)	0.0519 (14)	
H54	−0.0592	0.6069	0.0504	0.062*	
C55	−0.0581 (4)	0.6523 (3)	0.1554 (3)	0.0479 (12)	
H55	−0.1337	0.6622	0.1444	0.057*	
C56	0.0075 (3)	0.6712 (3)	0.2281 (2)	0.0383 (11)	
H56	−0.0243	0.6954	0.2659	0.046*	
C57	0.3560 (4)	0.8248 (3)	0.3485 (3)	0.0436 (11)	
H57	0.3877	0.7853	0.3159	0.052*	
C58	0.3943 (4)	0.9148 (3)	0.3754 (3)	0.0540 (13)	
H58	0.4510	0.9353	0.3610	0.065*	
C59	0.3506 (4)	0.9725 (3)	0.4218 (3)	0.0509 (13)	
H59	0.3770	1.0334	0.4402	0.061*	
C60	0.2657 (3)	0.9420 (3)	0.4428 (2)	0.0377 (10)	
C61	0.2327 (3)	0.8515 (3)	0.4135 (2)	0.0310 (9)	
C62	0.2158 (4)	0.9964 (3)	0.4924 (2)	0.0450 (12)	
H62	0.2395	1.0579	0.5135	0.054*	
C63	0.1353 (4)	0.9622 (3)	0.5098 (2)	0.0428 (11)	
H63	0.1036	0.9999	0.5434	0.051*	
C64	0.0968 (3)	0.8696 (3)	0.4785 (2)	0.0350 (10)	
C65	0.1474 (3)	0.8161 (3)	0.4315 (2)	0.0304 (9)	
C66	0.0108 (4)	0.8297 (3)	0.4916 (2)	0.0458 (12)	
H66	−0.0265	0.8639	0.5230	0.055*	
C67	−0.0190 (4)	0.7413 (3)	0.4589 (2)	0.0450 (12)	
H67	−0.0775	0.7135	0.4673	0.054*	
C68	0.0361 (3)	0.6915 (3)	0.4129 (2)	0.0379 (10)	
H68	0.0141	0.6298	0.3907	0.045*	
N21	0.9342 (4)	0.8805 (3)	0.3236 (3)	0.0737 (14)	
C70	0.6627 (4)	0.7663 (3)	0.3096 (3)	0.0519 (13)	
N20	0.6003 (4)	0.8084 (3)	0.3261 (3)	0.0895 (17)	
N19	0.9140 (5)	0.2258 (4)	0.3444 (3)	0.0921 (18)	
C69	0.7423 (4)	0.7123 (3)	0.2876 (3)	0.0703 (16)	
H69A	0.7475	0.6677	0.3082	0.106*	
H69B	0.8162	0.7487	0.3059	0.106*	
H69C	0.7175	0.6839	0.2336	0.106*	
C74	0.8294 (6)	0.2449 (4)	0.3437 (3)	0.078 (2)	
C75	0.7198 (6)	0.2697 (4)	0.3412 (4)	0.105 (3)	
H75A	0.6719	0.2521	0.2898	0.158*	
H75B	0.7285	0.3332	0.3672	0.158*	
H75C	0.6851	0.2405	0.3651	0.158*	
C78	0.9856 (6)	1.0175 (4)	0.3033 (3)	0.105 (3)	
H78A	1.0594	1.0493	0.3388	0.158*	
H78B	0.9288	1.0552	0.3099	0.158*	
H78C	0.9876	1.0002	0.2530	0.158*	
C77	0.9574 (4)	0.9401 (4)	0.3151 (3)	0.0577 (15)	
O1	0.0464 (10)	0.5593 (6)	0.5096 (4)	0.250 (5)	
H1O	0.065 (13)	0.589 (9)	0.5548 (17)	0.376*	
H2O	−0.001 (11)	0.585 (10)	0.490 (7)	0.376*	

### Atomic displacement parameters (Å^2^)

**Table:**

	*U*^11^	*U*^22^	*U*^33^	*U*^12^	*U*^13^	*U*^23^
Cr1	0.0235 (3)	0.0264 (4)	0.0375 (4)	0.0009 (3)	0.0060 (3)	0.0105 (3)
Cr2	0.0239 (3)	0.0303 (4)	0.0328 (4)	0.0069 (3)	0.0082 (3)	0.0142 (3)
Fe1	0.0257 (3)	0.0230 (3)	0.0286 (3)	0.0021 (2)	0.0103 (3)	0.0076 (3)
S1	0.0837 (10)	0.0521 (9)	0.0546 (8)	0.0133 (7)	0.0436 (8)	0.0232 (7)
S2	0.0506 (8)	0.0322 (7)	0.0728 (9)	−0.0058 (6)	0.0107 (7)	0.0243 (7)
S3	0.0345 (7)	0.0514 (8)	0.0649 (9)	0.0073 (6)	−0.0027 (6)	0.0289 (7)
S4	0.0887 (10)	0.0291 (7)	0.0478 (8)	0.0071 (6)	0.0297 (7)	0.0174 (6)
S5	0.0675 (9)	0.0559 (9)	0.0355 (7)	0.0033 (7)	0.0024 (6)	0.0060 (7)
S6	0.0362 (6)	0.0504 (8)	0.0618 (8)	0.0098 (5)	0.0209 (6)	0.0363 (7)
S7	0.0433 (7)	0.0339 (7)	0.0687 (9)	0.0040 (5)	0.0086 (6)	0.0272 (7)
S8	0.0311 (6)	0.0551 (8)	0.0519 (7)	0.0036 (5)	0.0177 (6)	0.0217 (6)
N1	0.033 (2)	0.036 (2)	0.037 (2)	0.0016 (16)	0.0077 (18)	0.0109 (19)
N2	0.031 (2)	0.030 (2)	0.046 (2)	0.0031 (16)	0.0094 (17)	0.0133 (19)
N3	0.029 (2)	0.034 (2)	0.035 (2)	0.0020 (16)	0.0022 (17)	0.0109 (18)
N4	0.0296 (19)	0.031 (2)	0.039 (2)	−0.0029 (16)	0.0026 (16)	0.0126 (18)
N5	0.036 (2)	0.038 (2)	0.034 (2)	0.0043 (17)	0.0089 (18)	0.0132 (19)
N6	0.0309 (19)	0.034 (2)	0.043 (2)	0.0087 (16)	0.0124 (17)	0.0190 (19)
N7	0.0314 (19)	0.036 (2)	0.034 (2)	0.0055 (16)	0.0067 (16)	0.0164 (18)
N8	0.0265 (19)	0.035 (2)	0.043 (2)	0.0063 (16)	0.0072 (17)	0.0175 (18)
N9	0.0203 (17)	0.030 (2)	0.038 (2)	0.0013 (15)	0.0074 (16)	0.0109 (17)
N10	0.0224 (17)	0.0243 (19)	0.034 (2)	0.0016 (14)	0.0069 (15)	0.0082 (16)
N11	0.0310 (19)	0.030 (2)	0.034 (2)	0.0117 (15)	0.0126 (16)	0.0151 (17)
N12	0.0289 (18)	0.0254 (19)	0.031 (2)	0.0070 (15)	0.0108 (16)	0.0124 (16)
N13	0.0271 (18)	0.030 (2)	0.032 (2)	0.0050 (15)	0.0102 (16)	0.0053 (17)
N14	0.0282 (18)	0.0273 (19)	0.0263 (18)	0.0027 (15)	0.0106 (15)	0.0081 (16)
N15	0.035 (2)	0.0222 (19)	0.039 (2)	0.0055 (15)	0.0183 (17)	0.0126 (17)
N16	0.035 (2)	0.0231 (19)	0.036 (2)	0.0023 (15)	0.0148 (17)	0.0134 (16)
N17	0.0312 (19)	0.0223 (19)	0.039 (2)	0.0005 (15)	0.0130 (17)	0.0078 (17)
N18	0.0296 (18)	0.033 (2)	0.0282 (19)	0.0064 (15)	0.0106 (15)	0.0138 (17)
C1	0.030 (2)	0.028 (2)	0.053 (3)	0.0021 (19)	0.017 (2)	0.016 (2)
C2	0.029 (2)	0.032 (3)	0.040 (3)	0.0062 (19)	0.009 (2)	0.017 (2)
C3	0.032 (2)	0.034 (3)	0.036 (3)	−0.003 (2)	0.005 (2)	0.013 (2)
C4	0.033 (2)	0.031 (3)	0.025 (2)	0.0026 (19)	0.0085 (18)	0.010 (2)
C5	0.031 (2)	0.036 (3)	0.044 (3)	0.008 (2)	0.010 (2)	0.021 (2)
C6	0.020 (2)	0.035 (3)	0.030 (2)	0.0070 (18)	0.0094 (18)	0.007 (2)
C7	0.020 (2)	0.038 (3)	0.029 (2)	0.0034 (18)	0.0046 (18)	0.016 (2)
C8	0.031 (2)	0.030 (2)	0.032 (2)	0.0085 (19)	0.0026 (19)	0.014 (2)
C9	0.026 (2)	0.048 (3)	0.047 (3)	0.006 (2)	0.010 (2)	0.014 (2)
C10	0.037 (3)	0.054 (3)	0.050 (3)	0.009 (2)	0.020 (2)	0.014 (3)
C11	0.041 (3)	0.037 (3)	0.042 (3)	0.000 (2)	0.016 (2)	0.010 (2)
C12	0.035 (2)	0.024 (2)	0.038 (3)	−0.0030 (19)	0.011 (2)	0.007 (2)
C13	0.024 (2)	0.022 (2)	0.037 (2)	−0.0023 (17)	0.0084 (19)	0.007 (2)
C14	0.042 (3)	0.033 (3)	0.038 (3)	−0.001 (2)	0.008 (2)	0.015 (2)
C15	0.033 (2)	0.034 (3)	0.047 (3)	0.004 (2)	0.004 (2)	0.023 (2)
C16	0.027 (2)	0.021 (2)	0.041 (3)	−0.0006 (17)	0.0040 (19)	0.012 (2)
C17	0.023 (2)	0.019 (2)	0.037 (2)	0.0018 (16)	0.0060 (18)	0.0090 (19)
C18	0.023 (2)	0.030 (3)	0.052 (3)	0.0047 (18)	0.005 (2)	0.018 (2)
C19	0.026 (2)	0.033 (3)	0.051 (3)	0.0045 (19)	0.014 (2)	0.011 (2)
C20	0.031 (2)	0.027 (2)	0.042 (3)	0.0032 (18)	0.013 (2)	0.008 (2)
C21	0.037 (3)	0.052 (3)	0.044 (3)	0.018 (2)	0.019 (2)	0.018 (3)
C22	0.053 (3)	0.069 (4)	0.040 (3)	0.027 (3)	0.026 (3)	0.017 (3)
C23	0.059 (3)	0.044 (3)	0.033 (3)	0.015 (2)	0.015 (2)	0.006 (2)
C24	0.042 (3)	0.031 (3)	0.032 (3)	0.007 (2)	0.009 (2)	0.012 (2)
C25	0.031 (2)	0.023 (2)	0.033 (2)	0.0071 (18)	0.0100 (19)	0.012 (2)
C26	0.045 (3)	0.028 (3)	0.035 (3)	0.002 (2)	0.000 (2)	0.007 (2)
C27	0.031 (2)	0.031 (3)	0.046 (3)	−0.0037 (19)	0.001 (2)	0.015 (2)
C28	0.029 (2)	0.023 (2)	0.040 (3)	0.0028 (18)	0.008 (2)	0.015 (2)
C29	0.029 (2)	0.020 (2)	0.030 (2)	0.0035 (17)	0.0094 (19)	0.0111 (19)
C30	0.023 (2)	0.033 (3)	0.049 (3)	0.0005 (19)	0.009 (2)	0.017 (2)
C31	0.032 (2)	0.042 (3)	0.052 (3)	0.012 (2)	0.024 (2)	0.022 (2)
C32	0.032 (2)	0.036 (3)	0.033 (2)	0.0055 (19)	0.013 (2)	0.011 (2)
C33	0.027 (2)	0.039 (3)	0.041 (3)	−0.003 (2)	0.007 (2)	0.003 (2)
C34	0.029 (3)	0.063 (4)	0.032 (3)	0.001 (2)	0.001 (2)	−0.001 (3)
C35	0.039 (3)	0.082 (4)	0.030 (3)	0.022 (3)	0.009 (2)	0.016 (3)
C36	0.039 (3)	0.055 (3)	0.033 (3)	0.019 (2)	0.012 (2)	0.014 (2)
C37	0.031 (2)	0.033 (3)	0.031 (2)	0.0106 (19)	0.0139 (19)	0.011 (2)
C38	0.061 (3)	0.064 (4)	0.045 (3)	0.033 (3)	0.020 (3)	0.035 (3)
C39	0.072 (4)	0.051 (3)	0.054 (3)	0.026 (3)	0.027 (3)	0.032 (3)
C40	0.059 (3)	0.036 (3)	0.036 (3)	0.013 (2)	0.023 (2)	0.017 (2)
C41	0.034 (2)	0.030 (2)	0.029 (2)	0.0047 (19)	0.0134 (19)	0.012 (2)
C42	0.070 (3)	0.031 (3)	0.047 (3)	−0.003 (2)	0.025 (3)	0.012 (2)
C43	0.058 (3)	0.034 (3)	0.033 (3)	−0.011 (2)	0.008 (2)	0.008 (2)
C44	0.044 (3)	0.029 (3)	0.032 (2)	−0.005 (2)	0.009 (2)	0.007 (2)
C45	0.036 (3)	0.026 (2)	0.061 (3)	0.0069 (19)	0.027 (2)	0.016 (2)
C46	0.051 (3)	0.031 (3)	0.094 (4)	0.013 (2)	0.048 (3)	0.023 (3)
C47	0.080 (4)	0.028 (3)	0.088 (4)	0.011 (3)	0.067 (4)	0.017 (3)
C48	0.066 (3)	0.027 (3)	0.055 (3)	0.004 (2)	0.041 (3)	0.012 (2)
C49	0.049 (3)	0.015 (2)	0.039 (3)	0.0032 (19)	0.026 (2)	0.0084 (19)
C50	0.106 (5)	0.043 (3)	0.055 (4)	0.011 (3)	0.053 (4)	0.014 (3)
C51	0.116 (5)	0.032 (3)	0.035 (3)	0.002 (3)	0.032 (3)	0.007 (2)
C52	0.079 (4)	0.021 (2)	0.031 (3)	−0.005 (2)	0.012 (3)	0.007 (2)
C53	0.051 (3)	0.020 (2)	0.035 (3)	0.002 (2)	0.019 (2)	0.009 (2)
C54	0.076 (4)	0.027 (3)	0.031 (3)	−0.011 (2)	−0.009 (3)	0.011 (2)
C55	0.050 (3)	0.038 (3)	0.050 (3)	−0.001 (2)	−0.001 (3)	0.026 (3)
C56	0.035 (3)	0.038 (3)	0.041 (3)	0.004 (2)	0.008 (2)	0.018 (2)
C57	0.043 (3)	0.030 (3)	0.064 (3)	0.006 (2)	0.030 (2)	0.016 (2)
C58	0.052 (3)	0.039 (3)	0.078 (4)	0.000 (2)	0.030 (3)	0.025 (3)
C59	0.051 (3)	0.024 (3)	0.073 (4)	0.003 (2)	0.020 (3)	0.015 (3)
C60	0.036 (2)	0.027 (3)	0.043 (3)	0.007 (2)	0.009 (2)	0.010 (2)
C61	0.031 (2)	0.026 (2)	0.033 (2)	0.0090 (18)	0.0069 (19)	0.011 (2)
C62	0.046 (3)	0.024 (3)	0.045 (3)	0.011 (2)	0.004 (2)	0.001 (2)
C63	0.046 (3)	0.039 (3)	0.033 (3)	0.019 (2)	0.011 (2)	0.003 (2)
C64	0.034 (2)	0.039 (3)	0.031 (2)	0.016 (2)	0.012 (2)	0.012 (2)
C65	0.030 (2)	0.028 (2)	0.031 (2)	0.0089 (18)	0.0063 (19)	0.012 (2)
C66	0.051 (3)	0.051 (3)	0.039 (3)	0.022 (2)	0.023 (2)	0.014 (3)
C67	0.045 (3)	0.053 (3)	0.051 (3)	0.013 (2)	0.031 (2)	0.024 (3)
C68	0.039 (3)	0.037 (3)	0.041 (3)	0.007 (2)	0.020 (2)	0.015 (2)
N21	0.066 (3)	0.083 (4)	0.068 (3)	−0.011 (3)	0.011 (3)	0.036 (3)
C70	0.042 (3)	0.050 (3)	0.065 (4)	0.000 (3)	0.014 (3)	0.030 (3)
N20	0.083 (4)	0.079 (4)	0.135 (5)	0.030 (3)	0.064 (4)	0.050 (4)
N19	0.100 (5)	0.093 (4)	0.077 (4)	0.000 (4)	0.052 (4)	0.011 (3)
C69	0.049 (3)	0.068 (4)	0.098 (5)	0.004 (3)	0.023 (3)	0.039 (4)
C74	0.106 (6)	0.053 (4)	0.079 (4)	−0.007 (4)	0.065 (5)	0.006 (3)
C75	0.132 (6)	0.056 (4)	0.161 (7)	0.021 (4)	0.109 (6)	0.032 (4)
C78	0.165 (7)	0.057 (4)	0.101 (5)	−0.020 (4)	0.084 (5)	0.011 (4)
C77	0.053 (3)	0.069 (4)	0.034 (3)	−0.014 (3)	0.013 (2)	0.005 (3)
O1	0.305 (13)	0.339 (13)	0.093 (5)	0.207 (11)	0.040 (7)	0.076 (9)

### Geometric parameters (Å, º)

**Table:**

Cr1—N4	1.969 (3)	C27—H27	0.9500
Cr1—N3	1.975 (3)	C28—C30	1.398 (5)
Cr1—N2	1.978 (4)	C28—C29	1.407 (5)
Cr1—N1	1.986 (4)	C30—C31	1.363 (6)
Cr1—N10	2.064 (3)	C30—H30	0.9500
Cr1—N9	2.066 (3)	C31—C32	1.393 (5)
Cr2—N7	1.963 (3)	C31—H31	0.9500
Cr2—N5	1.977 (4)	C32—H32	0.9500
Cr2—N8	1.984 (3)	C33—C34	1.392 (6)
Cr2—N6	1.999 (3)	C33—H33	0.9500
Cr2—N12	2.071 (3)	C34—C35	1.361 (6)
Cr2—N11	2.078 (3)	C34—H34	0.9500
Fe1—N16	1.959 (3)	C35—C36	1.402 (6)
Fe1—N15	1.972 (3)	C35—H35	0.9500
Fe1—N13	1.976 (3)	C36—C37	1.399 (5)
Fe1—N18	1.977 (3)	C36—C38	1.423 (6)
Fe1—N17	1.978 (3)	C37—C41	1.425 (5)
Fe1—N14	1.978 (3)	C38—C39	1.342 (6)
S1—C1	1.615 (5)	C38—H38	0.9500
S2—C2	1.616 (4)	C39—C40	1.424 (6)
S3—C3	1.608 (4)	C39—H39	0.9500
S4—C4	1.606 (4)	C40—C41	1.395 (5)
S5—C5	1.619 (5)	C40—C42	1.410 (6)
S6—C6	1.623 (4)	C42—C43	1.357 (6)
S7—C7	1.612 (4)	C42—H42	0.9500
S8—C8	1.616 (4)	C43—C44	1.382 (5)
N1—C1	1.168 (5)	C43—H43	0.9500
N2—C2	1.153 (5)	C44—H44	0.9500
N3—C3	1.163 (5)	C45—C46	1.397 (6)
N4—C4	1.166 (5)	C45—H45	0.9500
N5—C5	1.163 (5)	C46—C47	1.366 (7)
N6—C6	1.166 (5)	C46—H46	0.9500
N7—C7	1.160 (5)	C47—C48	1.396 (7)
N8—C8	1.166 (5)	C47—H47	0.9500
N9—C9	1.318 (5)	C48—C49	1.398 (6)
N9—C13	1.359 (5)	C48—C50	1.437 (7)
N10—C20	1.324 (5)	C49—C53	1.412 (6)
N10—C17	1.364 (5)	C50—C51	1.330 (7)
N11—C21	1.324 (5)	C50—H50	0.9500
N11—C25	1.368 (5)	C51—C52	1.432 (7)
N12—C32	1.324 (5)	C51—H51	0.9500
N12—C29	1.364 (5)	C52—C54	1.397 (7)
N13—C33	1.335 (5)	C52—C53	1.403 (6)
N13—C37	1.366 (5)	C54—C55	1.362 (6)
N14—C44	1.334 (5)	C54—H54	0.9500
N14—C41	1.361 (5)	C55—C56	1.394 (6)
N15—C45	1.327 (5)	C55—H55	0.9500
N15—C49	1.370 (5)	C56—H56	0.9500
N16—C56	1.327 (5)	C57—C58	1.403 (6)
N16—C53	1.370 (5)	C57—H57	0.9500
N17—C57	1.334 (5)	C58—C59	1.354 (6)
N17—C61	1.363 (5)	C58—H58	0.9500
N18—C68	1.335 (5)	C59—C60	1.412 (6)
N18—C65	1.368 (5)	C59—H59	0.9500
C9—C10	1.393 (6)	C60—C61	1.396 (5)
C9—H9	0.9500	C60—C62	1.421 (6)
C10—C11	1.373 (6)	C61—C65	1.415 (5)
C10—H10	0.9500	C62—C63	1.348 (6)
C11—C12	1.395 (6)	C62—H62	0.9500
C11—H11	0.9500	C63—C64	1.435 (6)
C12—C13	1.403 (5)	C63—H63	0.9500
C12—C14	1.427 (6)	C64—C65	1.390 (5)
C13—C17	1.422 (5)	C64—C66	1.396 (6)
C14—C15	1.347 (6)	C66—C67	1.358 (6)
C14—H14	0.9500	C66—H66	0.9500
C15—C16	1.442 (5)	C67—C68	1.393 (6)
C15—H15	0.9500	C67—H67	0.9500
C16—C18	1.386 (5)	C68—H68	0.9500
C16—C17	1.399 (5)	N21—C77	1.114 (6)
C18—C19	1.364 (6)	C70—N20	1.123 (6)
C18—H18	0.9500	C70—C69	1.440 (7)
C19—C20	1.400 (5)	N19—C74	1.125 (8)
C19—H19	0.9500	C69—H69A	0.9800
C20—H20	0.9500	C69—H69B	0.9800
C21—C22	1.398 (6)	C69—H69C	0.9800
C21—H21	0.9500	C74—C75	1.453 (9)
C22—C23	1.367 (6)	C75—H75A	0.9800
C22—H22	0.9500	C75—H75B	0.9800
C23—C24	1.392 (6)	C75—H75C	0.9800
C23—H23	0.9500	C78—C77	1.449 (7)
C24—C25	1.403 (5)	C78—H78A	0.9800
C24—C26	1.433 (6)	C78—H78B	0.9800
C25—C29	1.414 (5)	C78—H78C	0.9800
C26—C27	1.348 (6)	O1—H1O	0.84 (2)
C26—H26	0.9500	O1—H2O	0.85 (2)
C27—C28	1.439 (6)		
			
N4—Cr1—N3	93.08 (13)	C28—C27—H27	119.5
N4—Cr1—N2	174.49 (14)	C30—C28—C29	117.6 (4)
N3—Cr1—N2	91.37 (14)	C30—C28—C27	124.1 (4)
N4—Cr1—N1	91.16 (14)	C29—C28—C27	118.3 (4)
N3—Cr1—N1	93.32 (14)	N12—C29—C28	121.9 (4)
N2—Cr1—N1	91.82 (14)	N12—C29—C25	117.9 (3)
N4—Cr1—N10	87.71 (13)	C28—C29—C25	120.2 (4)
N3—Cr1—N10	173.14 (14)	C31—C30—C28	119.6 (4)
N2—Cr1—N10	87.50 (13)	C31—C30—H30	120.2
N1—Cr1—N10	93.48 (14)	C28—C30—H30	120.2
N4—Cr1—N9	88.10 (13)	C30—C31—C32	119.7 (4)
N3—Cr1—N9	93.48 (13)	C30—C31—H31	120.2
N2—Cr1—N9	88.38 (13)	C32—C31—H31	120.2
N1—Cr1—N9	173.20 (13)	N12—C32—C31	122.3 (4)
N10—Cr1—N9	79.73 (13)	N12—C32—H32	118.8
N7—Cr2—N5	89.11 (14)	C31—C32—H32	118.8
N7—Cr2—N8	89.61 (13)	N13—C33—C34	122.6 (4)
N5—Cr2—N8	94.14 (14)	N13—C33—H33	118.7
N7—Cr2—N6	175.77 (13)	C34—C33—H33	118.7
N5—Cr2—N6	90.40 (14)	C35—C34—C33	119.9 (4)
N8—Cr2—N6	94.62 (13)	C35—C34—H34	120.0
N7—Cr2—N12	88.04 (12)	C33—C34—H34	120.0
N5—Cr2—N12	95.01 (13)	C34—C35—C36	119.9 (4)
N8—Cr2—N12	170.52 (13)	C34—C35—H35	120.0
N6—Cr2—N12	87.81 (12)	C36—C35—H35	120.0
N7—Cr2—N11	90.64 (13)	C37—C36—C35	116.5 (4)
N5—Cr2—N11	174.81 (13)	C37—C36—C38	118.2 (4)
N8—Cr2—N11	91.05 (13)	C35—C36—C38	125.3 (4)
N6—Cr2—N11	89.46 (13)	N13—C37—C36	124.0 (4)
N12—Cr2—N11	79.80 (12)	N13—C37—C41	115.8 (4)
N16—Fe1—N15	82.94 (14)	C36—C37—C41	120.2 (4)
N16—Fe1—N13	173.04 (13)	C39—C38—C36	121.9 (4)
N15—Fe1—N13	92.81 (13)	C39—C38—H38	119.0
N16—Fe1—N18	95.12 (13)	C36—C38—H38	119.0
N15—Fe1—N18	173.96 (13)	C38—C39—C40	120.7 (5)
N13—Fe1—N18	89.65 (13)	C38—C39—H39	119.7
N16—Fe1—N17	92.39 (13)	C40—C39—H39	119.7
N15—Fe1—N17	92.11 (13)	C41—C40—C42	116.1 (4)
N13—Fe1—N17	93.27 (13)	C41—C40—C39	119.1 (4)
N18—Fe1—N17	82.24 (13)	C42—C40—C39	124.7 (4)
N16—Fe1—N14	92.28 (13)	N14—C41—C40	124.7 (4)
N15—Fe1—N14	93.11 (12)	N14—C41—C37	115.5 (3)
N13—Fe1—N14	82.42 (13)	C40—C41—C37	119.8 (4)
N18—Fe1—N14	92.68 (13)	C43—C42—C40	119.3 (4)
N17—Fe1—N14	173.38 (13)	C43—C42—H42	120.3
C1—N1—Cr1	166.9 (3)	C40—C42—H42	120.3
C2—N2—Cr1	176.1 (3)	C42—C43—C44	120.5 (4)
C3—N3—Cr1	170.3 (3)	C42—C43—H43	119.7
C4—N4—Cr1	170.4 (3)	C44—C43—H43	119.7
C5—N5—Cr2	167.4 (3)	N14—C44—C43	122.8 (4)
C6—N6—Cr2	166.9 (3)	N14—C44—H44	118.6
C7—N7—Cr2	169.4 (3)	C43—C44—H44	118.6
C8—N8—Cr2	170.1 (3)	N15—C45—C46	122.7 (5)
C9—N9—C13	118.1 (3)	N15—C45—H45	118.6
C9—N9—Cr1	128.4 (3)	C46—C45—H45	118.6
C13—N9—Cr1	113.5 (3)	C47—C46—C45	119.9 (5)
C20—N10—C17	118.6 (3)	C47—C46—H46	120.1
C20—N10—Cr1	128.2 (3)	C45—C46—H46	120.1
C17—N10—Cr1	113.2 (2)	C46—C47—C48	119.5 (4)
C21—N11—C25	117.5 (4)	C46—C47—H47	120.3
C21—N11—Cr2	129.3 (3)	C48—C47—H47	120.3
C25—N11—Cr2	113.1 (3)	C47—C48—C49	117.2 (5)
C32—N12—C29	118.8 (3)	C47—C48—C50	125.3 (5)
C32—N12—Cr2	128.5 (3)	C49—C48—C50	117.5 (5)
C29—N12—Cr2	112.7 (2)	N15—C49—C48	123.6 (4)
C33—N13—C37	117.0 (4)	N15—C49—C53	115.7 (4)
C33—N13—Fe1	130.0 (3)	C48—C49—C53	120.8 (4)
C37—N13—Fe1	113.0 (3)	C51—C50—C48	121.9 (5)
C44—N14—C41	116.5 (3)	C51—C50—H50	119.1
C44—N14—Fe1	130.2 (3)	C48—C50—H50	119.1
C41—N14—Fe1	113.3 (2)	C50—C51—C52	121.6 (5)
C45—N15—C49	117.1 (4)	C50—C51—H51	119.2
C45—N15—Fe1	130.3 (3)	C52—C51—H51	119.2
C49—N15—Fe1	112.6 (3)	C54—C52—C53	117.1 (4)
C56—N16—C53	117.1 (4)	C54—C52—C51	125.2 (5)
C56—N16—Fe1	129.9 (3)	C53—C52—C51	117.7 (5)
C53—N16—Fe1	112.9 (3)	N16—C53—C52	123.6 (4)
C57—N17—C61	117.1 (4)	N16—C53—C49	115.9 (4)
C57—N17—Fe1	130.0 (3)	C52—C53—C49	120.6 (4)
C61—N17—Fe1	112.7 (3)	C55—C54—C52	119.2 (4)
C68—N18—C65	117.0 (3)	C55—C54—H54	120.4
C68—N18—Fe1	129.8 (3)	C52—C54—H54	120.4
C65—N18—Fe1	113.0 (2)	C54—C55—C56	120.4 (5)
N1—C1—S1	178.2 (4)	C54—C55—H55	119.8
N2—C2—S2	178.8 (4)	C56—C55—H55	119.8
N3—C3—S3	179.4 (4)	N16—C56—C55	122.6 (4)
N4—C4—S4	178.2 (4)	N16—C56—H56	118.7
N5—C5—S5	179.2 (4)	C55—C56—H56	118.7
N6—C6—S6	178.1 (3)	N17—C57—C58	122.1 (4)
N7—C7—S7	179.2 (4)	N17—C57—H57	118.9
N8—C8—S8	178.8 (4)	C58—C57—H57	118.9
N9—C9—C10	122.6 (4)	C59—C58—C57	120.2 (4)
N9—C9—H9	118.7	C59—C58—H58	119.9
C10—C9—H9	118.7	C57—C58—H58	119.9
C11—C10—C9	119.5 (4)	C58—C59—C60	119.9 (4)
C11—C10—H10	120.2	C58—C59—H59	120.1
C9—C10—H10	120.2	C60—C59—H59	120.1
C10—C11—C12	119.5 (4)	C61—C60—C59	116.1 (4)
C10—C11—H11	120.2	C61—C60—C62	119.2 (4)
C12—C11—H11	120.2	C59—C60—C62	124.6 (4)
C11—C12—C13	117.0 (4)	N17—C61—C60	124.5 (4)
C11—C12—C14	124.5 (4)	N17—C61—C65	116.1 (4)
C13—C12—C14	118.4 (4)	C60—C61—C65	119.4 (4)
N9—C13—C12	123.2 (4)	C63—C62—C60	121.1 (4)
N9—C13—C17	116.6 (3)	C63—C62—H62	119.5
C12—C13—C17	120.2 (4)	C60—C62—H62	119.5
C15—C14—C12	121.6 (4)	C62—C63—C64	121.0 (4)
C15—C14—H14	119.2	C62—C63—H63	119.5
C12—C14—H14	119.2	C64—C63—H63	119.5
C14—C15—C16	121.1 (4)	C65—C64—C66	117.6 (4)
C14—C15—H15	119.5	C65—C64—C63	118.1 (4)
C16—C15—H15	119.5	C66—C64—C63	124.3 (4)
C18—C16—C17	117.2 (4)	N18—C65—C64	123.4 (4)
C18—C16—C15	124.6 (4)	N18—C65—C61	115.4 (3)
C17—C16—C15	118.1 (4)	C64—C65—C61	121.2 (4)
N10—C17—C16	122.6 (4)	C67—C66—C64	119.3 (4)
N10—C17—C13	116.9 (3)	C67—C66—H66	120.3
C16—C17—C13	120.6 (4)	C64—C66—H66	120.3
C19—C18—C16	120.4 (4)	C66—C67—C68	120.1 (4)
C19—C18—H18	119.8	C66—C67—H67	119.9
C16—C18—H18	119.8	C68—C67—H67	119.9
C18—C19—C20	119.3 (4)	N18—C68—C67	122.5 (4)
C18—C19—H19	120.4	N18—C68—H68	118.7
C20—C19—H19	120.4	C67—C68—H68	118.7
N10—C20—C19	122.0 (4)	N20—C70—C69	179.3 (6)
N10—C20—H20	119.0	C70—C69—H69A	109.5
C19—C20—H20	119.0	C70—C69—H69B	109.5
N11—C21—C22	123.1 (4)	H69A—C69—H69B	109.5
N11—C21—H21	118.5	C70—C69—H69C	109.5
C22—C21—H21	118.5	H69A—C69—H69C	109.5
C23—C22—C21	119.4 (4)	H69B—C69—H69C	109.5
C23—C22—H22	120.3	N19—C74—C75	178.8 (8)
C21—C22—H22	120.3	C74—C75—H75A	109.5
C22—C23—C24	119.5 (4)	C74—C75—H75B	109.5
C22—C23—H23	120.3	H75A—C75—H75B	109.5
C24—C23—H23	120.3	C74—C75—H75C	109.5
C23—C24—C25	117.8 (4)	H75A—C75—H75C	109.5
C23—C24—C26	124.2 (4)	H75B—C75—H75C	109.5
C25—C24—C26	117.9 (4)	C77—C78—H78A	109.5
N11—C25—C24	122.7 (4)	C77—C78—H78B	109.5
N11—C25—C29	116.4 (4)	H78A—C78—H78B	109.5
C24—C25—C29	120.9 (4)	C77—C78—H78C	109.5
C27—C26—C24	121.7 (4)	H78A—C78—H78C	109.5
C27—C26—H26	119.2	H78B—C78—H78C	109.5
C24—C26—H26	119.2	N21—C77—C78	179.0 (7)
C26—C27—C28	121.0 (4)	H1O—O1—H2O	105 (5)
C26—C27—H27	119.5		
			
N4—Cr1—N1—C1	−65.0 (15)	C20—N10—C17—C16	0.0 (5)
N3—Cr1—N1—C1	28.2 (15)	Cr1—N10—C17—C16	179.7 (3)
N2—Cr1—N1—C1	119.7 (15)	C20—N10—C17—C13	−178.1 (3)
N10—Cr1—N1—C1	−152.7 (15)	Cr1—N10—C17—C13	1.6 (4)
N9—Cr1—N1—C1	−148.7 (13)	C18—C16—C17—N10	−0.7 (5)
N4—Cr1—N2—C2	−30 (6)	C15—C16—C17—N10	−179.6 (3)
N3—Cr1—N2—C2	−173 (5)	C18—C16—C17—C13	177.3 (3)
N1—Cr1—N2—C2	93 (5)	C15—C16—C17—C13	−1.6 (5)
N10—Cr1—N2—C2	0 (5)	N9—C13—C17—N10	−0.4 (5)
N9—Cr1—N2—C2	−80 (5)	C12—C13—C17—N10	178.7 (3)
N4—Cr1—N3—C3	104 (2)	N9—C13—C17—C16	−178.5 (3)
N2—Cr1—N3—C3	−79 (2)	C12—C13—C17—C16	0.6 (5)
N1—Cr1—N3—C3	13 (2)	C17—C16—C18—C19	1.4 (6)
N10—Cr1—N3—C3	−159.7 (17)	C15—C16—C18—C19	−179.9 (4)
N9—Cr1—N3—C3	−168 (2)	C16—C18—C19—C20	−1.3 (6)
N3—Cr1—N4—C4	177 (2)	C17—N10—C20—C19	0.1 (5)
N2—Cr1—N4—C4	33 (3)	Cr1—N10—C20—C19	−179.5 (3)
N1—Cr1—N4—C4	−90 (2)	C18—C19—C20—N10	0.5 (6)
N10—Cr1—N4—C4	4 (2)	C25—N11—C21—C22	−1.5 (6)
N9—Cr1—N4—C4	83 (2)	Cr2—N11—C21—C22	179.1 (3)
N7—Cr2—N5—C5	−45.6 (16)	N11—C21—C22—C23	0.3 (7)
N8—Cr2—N5—C5	43.9 (16)	C21—C22—C23—C24	1.9 (7)
N6—Cr2—N5—C5	138.6 (16)	C22—C23—C24—C25	−2.6 (6)
N12—Cr2—N5—C5	−133.6 (16)	C22—C23—C24—C26	177.0 (4)
N11—Cr2—N5—C5	−132.9 (16)	C21—N11—C25—C24	0.7 (5)
N7—Cr2—N6—C6	−61 (3)	Cr2—N11—C25—C24	−179.9 (3)
N5—Cr2—N6—C6	22.6 (14)	C21—N11—C25—C29	−177.9 (3)
N8—Cr2—N6—C6	116.8 (14)	Cr2—N11—C25—C29	1.6 (4)
N12—Cr2—N6—C6	−72.4 (14)	C23—C24—C25—N11	1.4 (6)
N11—Cr2—N6—C6	−152.2 (14)	C26—C24—C25—N11	−178.2 (3)
N5—Cr2—N7—C7	−9.2 (17)	C23—C24—C25—C29	179.9 (4)
N8—Cr2—N7—C7	−103.3 (17)	C26—C24—C25—C29	0.3 (6)
N6—Cr2—N7—C7	74 (3)	C23—C24—C26—C27	−178.2 (4)
N12—Cr2—N7—C7	85.8 (17)	C25—C24—C26—C27	1.4 (6)
N11—Cr2—N7—C7	165.6 (17)	C24—C26—C27—C28	−1.7 (6)
N7—Cr2—N8—C8	−11 (2)	C26—C27—C28—C30	178.5 (4)
N5—Cr2—N8—C8	−100 (2)	C26—C27—C28—C29	0.3 (6)
N6—Cr2—N8—C8	169 (2)	C32—N12—C29—C28	0.6 (5)
N12—Cr2—N8—C8	65 (2)	Cr2—N12—C29—C28	−177.9 (3)
N11—Cr2—N8—C8	80 (2)	C32—N12—C29—C25	179.0 (3)
N4—Cr1—N9—C9	93.9 (3)	Cr2—N12—C29—C25	0.5 (4)
N3—Cr1—N9—C9	0.9 (3)	C30—C28—C29—N12	1.3 (5)
N2—Cr1—N9—C9	−90.4 (3)	C27—C28—C29—N12	179.6 (3)
N1—Cr1—N9—C9	177.8 (10)	C30—C28—C29—C25	−177.0 (3)
N10—Cr1—N9—C9	−178.1 (4)	C27—C28—C29—C25	1.3 (5)
N4—Cr1—N9—C13	−86.6 (3)	N11—C25—C29—N12	−1.4 (5)
N3—Cr1—N9—C13	−179.6 (3)	C24—C25—C29—N12	−180.0 (3)
N2—Cr1—N9—C13	89.2 (3)	N11—C25—C29—C28	177.0 (3)
N1—Cr1—N9—C13	−2.7 (12)	C24—C25—C29—C28	−1.5 (5)
N10—Cr1—N9—C13	1.4 (2)	C29—C28—C30—C31	−2.1 (6)
N4—Cr1—N10—C20	−93.5 (3)	C27—C28—C30—C31	179.7 (4)
N3—Cr1—N10—C20	169.8 (10)	C28—C30—C31—C32	1.0 (6)
N2—Cr1—N10—C20	89.2 (3)	C29—N12—C32—C31	−1.8 (6)
N1—Cr1—N10—C20	−2.5 (3)	Cr2—N12—C32—C31	176.5 (3)
N9—Cr1—N10—C20	178.0 (3)	C30—C31—C32—N12	1.0 (6)
N4—Cr1—N10—C17	86.9 (3)	C37—N13—C33—C34	1.2 (6)
N3—Cr1—N10—C17	−9.8 (12)	Fe1—N13—C33—C34	−179.2 (3)
N2—Cr1—N10—C17	−90.4 (3)	N13—C33—C34—C35	−0.2 (7)
N1—Cr1—N10—C17	177.9 (3)	C33—C34—C35—C36	−0.7 (7)
N9—Cr1—N10—C17	−1.6 (2)	C34—C35—C36—C37	0.5 (6)
N7—Cr2—N11—C21	90.5 (3)	C34—C35—C36—C38	−177.8 (4)
N5—Cr2—N11—C21	178 (61)	C33—N13—C37—C36	−1.4 (6)
N8—Cr2—N11—C21	0.8 (3)	Fe1—N13—C37—C36	179.0 (3)
N6—Cr2—N11—C21	−93.8 (3)	C33—N13—C37—C41	178.1 (3)
N12—Cr2—N11—C21	178.4 (4)	Fe1—N13—C37—C41	−1.5 (4)
N7—Cr2—N11—C25	−88.9 (3)	C35—C36—C37—N13	0.6 (6)
N5—Cr2—N11—C25	−1.7 (16)	C38—C36—C37—N13	179.0 (4)
N8—Cr2—N11—C25	−178.5 (3)	C35—C36—C37—C41	−179.0 (4)
N6—Cr2—N11—C25	86.8 (3)	C38—C36—C37—C41	−0.5 (6)
N12—Cr2—N11—C25	−1.0 (2)	C37—C36—C38—C39	−1.0 (7)
N7—Cr2—N12—C32	−87.0 (3)	C35—C36—C38—C39	177.3 (5)
N5—Cr2—N12—C32	1.9 (3)	C36—C38—C39—C40	1.2 (7)
N8—Cr2—N12—C32	−162.8 (7)	C38—C39—C40—C41	0.2 (7)
N6—Cr2—N12—C32	92.1 (3)	C38—C39—C40—C42	−177.8 (5)
N11—Cr2—N12—C32	−178.0 (3)	C44—N14—C41—C40	0.0 (6)
N7—Cr2—N12—C29	91.3 (3)	Fe1—N14—C41—C40	178.7 (3)
N5—Cr2—N12—C29	−179.7 (3)	C44—N14—C41—C37	−178.5 (3)
N8—Cr2—N12—C29	15.6 (9)	Fe1—N14—C41—C37	0.2 (4)
N6—Cr2—N12—C29	−89.5 (3)	C42—C40—C41—N14	−2.0 (6)
N11—Cr2—N12—C29	0.3 (2)	C39—C40—C41—N14	179.8 (4)
N16—Fe1—N13—C33	141.1 (10)	C42—C40—C41—C37	176.5 (4)
N15—Fe1—N13—C33	88.9 (4)	C39—C40—C41—C37	−1.7 (6)
N18—Fe1—N13—C33	−85.6 (4)	N13—C37—C41—N14	0.9 (5)
N17—Fe1—N13—C33	−3.4 (4)	C36—C37—C41—N14	−179.5 (4)
N14—Fe1—N13—C33	−178.3 (4)	N13—C37—C41—C40	−177.7 (4)
N16—Fe1—N13—C37	−39.3 (12)	C36—C37—C41—C40	1.9 (6)
N15—Fe1—N13—C37	−91.5 (3)	C41—C40—C42—C43	1.6 (6)
N18—Fe1—N13—C37	94.0 (3)	C39—C40—C42—C43	179.7 (4)
N17—Fe1—N13—C37	176.2 (3)	C40—C42—C43—C44	0.5 (7)
N14—Fe1—N13—C37	1.2 (3)	C41—N14—C44—C43	2.4 (6)
N16—Fe1—N14—C44	−6.8 (4)	Fe1—N14—C44—C43	−176.0 (3)
N15—Fe1—N14—C44	−89.9 (4)	C42—C43—C44—N14	−2.7 (7)
N13—Fe1—N14—C44	177.7 (4)	C49—N15—C45—C46	−1.6 (5)
N18—Fe1—N14—C44	88.4 (3)	Fe1—N15—C45—C46	−179.3 (3)
N17—Fe1—N14—C44	128.1 (11)	N15—C45—C46—C47	1.9 (6)
N16—Fe1—N14—C41	174.7 (3)	C45—C46—C47—C48	−1.3 (7)
N15—Fe1—N14—C41	91.7 (3)	C46—C47—C48—C49	0.5 (6)
N13—Fe1—N14—C41	−0.7 (3)	C46—C47—C48—C50	−179.8 (4)
N18—Fe1—N14—C41	−90.0 (3)	C45—N15—C49—C48	0.8 (5)
N17—Fe1—N14—C41	−50.4 (13)	Fe1—N15—C49—C48	178.9 (3)
N16—Fe1—N15—C45	177.4 (3)	C45—N15—C49—C53	−178.8 (3)
N13—Fe1—N15—C45	−8.1 (3)	Fe1—N15—C49—C53	−0.8 (4)
N18—Fe1—N15—C45	105.9 (13)	C47—C48—C49—N15	−0.2 (6)
N17—Fe1—N15—C45	85.3 (3)	C50—C48—C49—N15	−180.0 (4)
N14—Fe1—N15—C45	−90.6 (3)	C47—C48—C49—C53	179.4 (4)
N16—Fe1—N15—C49	−0.3 (2)	C50—C48—C49—C53	−0.4 (6)
N13—Fe1—N15—C49	174.2 (3)	C47—C48—C50—C51	179.6 (5)
N18—Fe1—N15—C49	−71.9 (14)	C49—C48—C50—C51	−0.7 (7)
N17—Fe1—N15—C49	−92.4 (3)	C48—C50—C51—C52	0.6 (8)
N14—Fe1—N15—C49	91.6 (3)	C50—C51—C52—C54	−178.4 (5)
N15—Fe1—N16—C56	178.0 (3)	C50—C51—C52—C53	0.6 (7)
N13—Fe1—N16—C56	125.3 (10)	C56—N16—C53—C52	1.5 (5)
N18—Fe1—N16—C56	−7.8 (3)	Fe1—N16—C53—C52	178.6 (3)
N17—Fe1—N16—C56	−90.2 (3)	C56—N16—C53—C49	−179.2 (3)
N14—Fe1—N16—C56	85.1 (3)	Fe1—N16—C53—C49	−2.1 (4)
N15—Fe1—N16—C53	1.3 (2)	C54—C52—C53—N16	−3.4 (6)
N13—Fe1—N16—C53	−51.4 (11)	C51—C52—C53—N16	177.5 (4)
N18—Fe1—N16—C53	175.6 (2)	C54—C52—C53—C49	177.4 (4)
N17—Fe1—N16—C53	93.2 (3)	C51—C52—C53—C49	−1.7 (6)
N14—Fe1—N16—C53	−91.5 (3)	N15—C49—C53—N16	1.9 (5)
N16—Fe1—N17—C57	−83.2 (4)	C48—C49—C53—N16	−177.7 (3)
N15—Fe1—N17—C57	−0.2 (4)	N15—C49—C53—C52	−178.8 (3)
N13—Fe1—N17—C57	92.7 (4)	C48—C49—C53—C52	1.6 (6)
N18—Fe1—N17—C57	−178.1 (4)	C53—C52—C54—C55	2.5 (6)
N14—Fe1—N17—C57	141.9 (11)	C51—C52—C54—C55	−178.4 (4)
N16—Fe1—N17—C61	101.1 (3)	C52—C54—C55—C56	−0.1 (6)
N15—Fe1—N17—C61	−175.9 (3)	C53—N16—C56—C55	1.1 (6)
N13—Fe1—N17—C61	−83.0 (3)	Fe1—N16—C56—C55	−175.4 (3)
N18—Fe1—N17—C61	6.2 (3)	C54—C55—C56—N16	−1.8 (6)
N14—Fe1—N17—C61	−33.8 (13)	C61—N17—C57—C58	0.5 (6)
N16—Fe1—N18—C68	86.4 (3)	Fe1—N17—C57—C58	−175.1 (3)
N15—Fe1—N18—C68	157.4 (12)	N17—C57—C58—C59	−0.1 (7)
N13—Fe1—N18—C68	−88.5 (3)	C57—C58—C59—C60	−0.4 (7)
N17—Fe1—N18—C68	178.1 (4)	C58—C59—C60—C61	0.5 (7)
N14—Fe1—N18—C68	−6.1 (3)	C58—C59—C60—C62	178.5 (5)
N16—Fe1—N18—C65	−98.4 (3)	C57—N17—C61—C60	−0.4 (6)
N15—Fe1—N18—C65	−27.5 (14)	Fe1—N17—C61—C60	175.9 (3)
N13—Fe1—N18—C65	86.6 (3)	C57—N17—C61—C65	179.0 (4)
N17—Fe1—N18—C65	−6.7 (2)	Fe1—N17—C61—C65	−4.8 (4)
N14—Fe1—N18—C65	169.0 (3)	C59—C60—C61—N17	−0.1 (6)
Cr1—N1—C1—S1	−6 (14)	C62—C60—C61—N17	−178.2 (4)
Cr1—N2—C2—S2	108 (22)	C59—C60—C61—C65	−179.4 (4)
Cr1—N3—C3—S3	−12 (36)	C62—C60—C61—C65	2.4 (6)
Cr1—N4—C4—S4	164 (11)	C61—C60—C62—C63	−1.8 (6)
Cr2—N5—C5—S5	−173 (100)	C59—C60—C62—C63	−179.8 (4)
Cr2—N6—C6—S6	−102 (11)	C60—C62—C63—C64	−0.5 (7)
Cr2—N7—C7—S7	−167 (100)	C62—C63—C64—C65	2.0 (6)
Cr2—N8—C8—S8	−146 (16)	C62—C63—C64—C66	−177.3 (4)
C13—N9—C9—C10	0.0 (6)	C68—N18—C65—C64	1.3 (5)
Cr1—N9—C9—C10	179.5 (3)	Fe1—N18—C65—C64	−174.5 (3)
N9—C9—C10—C11	0.6 (7)	C68—N18—C65—C61	−178.1 (3)
C9—C10—C11—C12	−0.8 (6)	Fe1—N18—C65—C61	6.1 (4)
C10—C11—C12—C13	0.4 (6)	C66—C64—C65—N18	−1.3 (6)
C10—C11—C12—C14	−179.0 (4)	C63—C64—C65—N18	179.3 (3)
C9—N9—C13—C12	−0.5 (5)	C66—C64—C65—C61	178.0 (4)
Cr1—N9—C13—C12	179.9 (3)	C63—C64—C65—C61	−1.3 (6)
C9—N9—C13—C17	178.6 (3)	N17—C61—C65—N18	−0.9 (5)
Cr1—N9—C13—C17	−1.0 (4)	C60—C61—C65—N18	178.5 (3)
C11—C12—C13—N9	0.3 (6)	N17—C61—C65—C64	179.7 (4)
C14—C12—C13—N9	179.7 (3)	C60—C61—C65—C64	−0.9 (6)
C11—C12—C13—C17	−178.7 (3)	C65—C64—C66—C67	0.6 (6)
C14—C12—C13—C17	0.7 (5)	C63—C64—C66—C67	179.9 (4)
C11—C12—C14—C15	178.5 (4)	C64—C66—C67—C68	0.1 (7)
C13—C12—C14—C15	−0.9 (6)	C65—N18—C68—C67	−0.5 (6)
C12—C14—C15—C16	−0.1 (6)	Fe1—N18—C68—C67	174.5 (3)
C14—C15—C16—C18	−177.4 (4)	C66—C67—C68—N18	−0.2 (7)
C14—C15—C16—C17	1.4 (6)		

## References

[bb1] Alonso, C., Ballster, L., Gutierrez, F., Rerpinaqn, M. F., Sanchez, A. E. & Azcondo, M. T. (2005). *Eur. J. Inorg. Chem* pp. 486–495.

[bb2] Bruker (2005). *APEX2*, *SAINT* and *SADABS.* Bruker AXS Inc., Madison, Wisconsin, USA.

[bb3] Cherkasova, T. G. & Gorunova, I. P. (2003). *Zh. Neorg. Khim* **48**, 611–615.

[bb4] Cucos, A., Avarvari, N., Andruh, M., Journaux, Y., Muller, A. & Schmidtmann, M. (2006). *Eur. J. Inorg. Chem* pp. 903–907.

[bb5] Kolotilov, S. V., Cador, O., Gavrilenko, K. S., Golhen, S., Ouahab, L. & Pavlishchuk, V. V. (2010). *Eur. J. Inorg. Chem* **8**, 1255–1266.

[bb6] Makhankova, V. G. (2011). *Glob. J. Inorg. Chem* **2**, 265–285.

[bb7] Nikitina, V. M., Nesterova, O. V., Kokozay, V. N., Dyakonenko, V. V., Shishkin, O. V. & Jezierska, J. (2009). *Polyhedron*, **28**, 1265–1272.

[bb8] Semenaka, V. V., Nesterova, O. V., Kokozay, V. N., Zybatyuk, R. I. & Shishkin, O. V. (2011). *Acta Cryst.* E**67**, m1021–m1022. 10.1107/S1600536811024998 PMC3212116 22090818

[bb9] Sheldrick, G. M. (2008). *Acta Cryst.* A**64**, 112–122. 10.1107/S0108767307043930 18156677

[bb10] Spek, A. L. (2009). *Acta Cryst.* D**65**, 148–155. 10.1107/S090744490804362X PMC2631630 19171970

[bb11] Westrip, S. P. (2010). *J. Appl. Cryst.* **43**, 920–925.

[bb12] Zhang, K.-L., Chen, W., Xu, Y., Wang, Z., Zhong, Z. J. & You, X.-Z. (2001). *Polyhedron*, **20**, 2033–2036.

